# SARS-CoV-2 diagnostic testing rates determine the sensitivity of genomic surveillance programs

**DOI:** 10.1038/s41588-022-01267-w

**Published:** 2023-01-09

**Authors:** Alvin X. Han, Amy Toporowski, Jilian A. Sacks, Mark D. Perkins, Sylvie Briand, Maria van Kerkhove, Emma Hannay, Sergio Carmona, Bill Rodriguez, Edyth Parker, Brooke E. Nichols, Colin A. Russell

**Affiliations:** 1 Department of Medical Microbiology and Infection Prevention, Amsterdam University Medical Center, University of Amsterdam, Amsterdam, the Netherlands; 2 Foundation for Innovative New Diagnostics (FIND), Geneva, Switzerland; 3 Department of Epidemic and Pandemic Preparedness and Prevention, Emergency Preparedness Programme, World Health Organization, Geneva, Switzerland; 4 Department of Immunology and Microbiology, The Scripps Research Institute, La Jolla, CA USA; 5 Department of Global Health, School of Public Health, Boston University, Boston, MA USA

**Keywords:** SARS-CoV-2, Microbial genetics

## Abstract

The first step in SARS-CoV-2 genomic surveillance is testing to identify people who are infected. However, global testing rates are falling as we emerge from the acute health emergency and remain low in many low- and middle-income countries (mean = 27 tests per 100,000 people per day). We simulated COVID-19 epidemics in a prototypical low- and middle-income country to investigate how testing rates, sampling strategies and sequencing proportions jointly impact surveillance outcomes, and showed that low testing rates and spatiotemporal biases delay time to detection of new variants by weeks to months and can lead to unreliable estimates of variant prevalence, even when the proportion of samples sequenced is increased. Accordingly, investments in wider access to diagnostics to support testing rates of approximately 100 tests per 100,000 people per day could enable more timely detection of new variants and reliable estimates of variant prevalence. The performance of global SARS-CoV-2 genomic surveillance programs is fundamentally limited by access to diagnostic testing.

## Abstract (web summary)

The PATAT model is used to simulate SARS-CoV-2 epidemics in low- and middle-income countries, finding that diagnostic testing rates and proportions of viruses sequenced underpin timely and accurate novel variant virus detection.

## Main

Since the start of the COVID-19 pandemic in 2019, unprecedented expansion of genomic surveillance efforts has led to the generation of more than 10 million SARS-CoV-2 sequences deposited in the publicly accessible Global Initiative on Sharing Avian Influenza Data database (https://www.gisaid.org/) as of May 2022. These efforts have been integral to understanding the COVID-19 pandemic^1^, including the identification of the Alpha variant in the United Kingdom during the fall of 2020^2^, the Delta variant in India in late 2020^3^ and the Omicron variant in southern Africa in November 2021^4^. Despite the value of these efforts for monitoring the evolution of SARS-CoV-2, the intensity of genomic surveillance is highly heterogeneous across countries. High-income countries (HICs), on average, produced 16 times more SARS-CoV-2 sequences per reported case than low- and middle-income countries (LMICs) as a result of long-standing socioeconomic inequalities and consequent underfunding of laboratory and surveillance infrastructures in LMICs^5^. To strengthen global pandemic preparedness, initiatives such as the Access to COVID-19 Tools Accelerator Global Risk Monitoring Framework, the Pan American Health Organization COVID-19 Genomic Surveillance Regional Network, the Africa Pathogen Genomics Initiative and the Global Influenza Surveillance and Response System, among others, have supported LMICs in developing pathogen genomic surveillance programs.

**Fig. 1:**
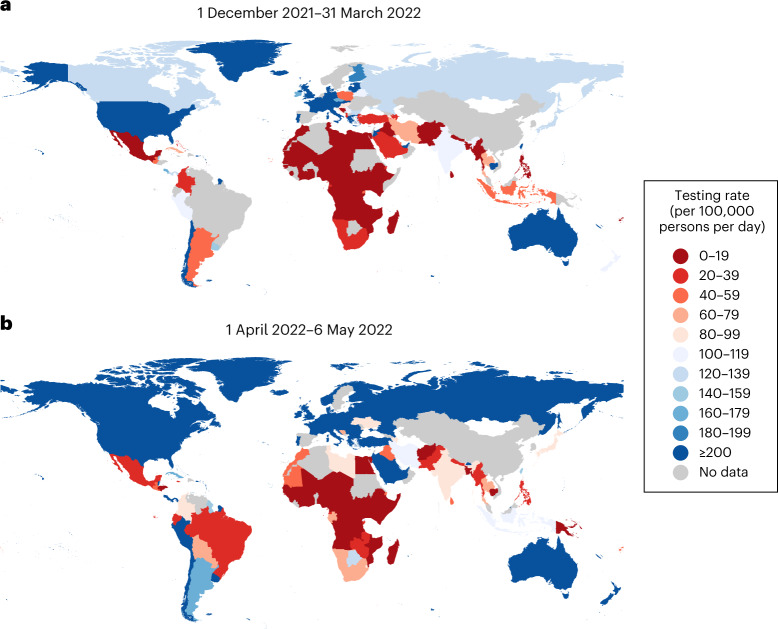
Global disparities in SARS-CoV-2 testing rates. **a**,**b**, The color of each country represents the average total number of SARS-CoV-2 tests performed per 100,000 persons per day between 1 December 2021 and 31 March 2022 when the Omicron VOC spread around the world (**a**), and between 1 April 2022 and 6 May 2022 when most countries were past peak Omicron wave of infections (**b**)^9^. Raster map from naturalearthdata.com.

Because resources are finite, it is critical that sequencing sample sizes, and the diagnostic testing needed to obtain samples for sequencing, are carefully set for genomic surveillance programs to detect and monitor variants as efficiently as possible. Current recommended sample sizes are based on sampling theory^5–8^ and assume that the volume of diagnostic testing is large enough such that the diversity of sampled viruses is representative of the diversity of viruses circulating in the population. However, LMICs test at a mean rate of 27 tests per 100,000 persons per day, as opposed to more than 800 tests per 100,000 people per day across HICs, based on observational data collected between January 2020 and March 2022^9^, with even higher testing rates in some HICs (Fig. 1). Low testing rates lead to spotty information and smaller virus specimen pools available for sequencing, resulting in sampling biases. These factors can render efforts to monitor the emergence of new SARS-CoV-2 variants or prevalence of existing variants highly unreliable.

Here, we studied how different testing rates can impact genomic surveillance outcomes. Specifically, we developed and used the Propelling Action for Testing And Treating (PATAT) model, an individual-based modeling framework, to simulate concurrently circulating wild-type SARS-CoV-2 (pre-Alpha viruses)/Alpha-like epidemics and Delta-/Omicron (BA.1)-like epidemics in Zambia as a representative LMIC archetype where recent demographic census data required by the model was available (Methods). We assumed that Alpha and Omicron (BA.1) were more transmissible than the respective extant virus to achieve growth rates of approximately 0.15 and 0.35 per day, respectively^2,10^, and simulated SARS-CoV-2 infection waves in a population of 1,000,000 individuals over a 90-day period that begins with an initial 1% prevalence of the extant SARS-CoV-2 variant and the mutant variant being introduced at 0.01%. We assumed that clinic-based, professional-use antigen rapid diagnostic tests (Ag-RDTs) form the basis of testing, given persistent reports that polymerase chain reaction (PCR) tests are poorly accessible for detection of individuals with COVID-19 symptoms outside of tertiary medical facilities (for example, inpatient hospitals with personnel and facilities for advanced medical investigation and treatment) in many LMICs^11^.

We then simulated different genomic surveillance sampling strategies to elucidate how testing, sequencing volumes and the degree of sampling bias arising from sources of specimens jointly impact the timeliness of variant detection and the accuracy of variant monitoring (Methods). These strategies include: (1) sending all samples from community clinics and tertiary hospitals to a centralized facility for possible sequencing (that is, population-wide strategy); (2) sampling and sequencing a portion of positive specimens collected at one tertiary sentinel facility for the population of 1,000,000 simulated people (including individuals with mild symptoms seeking symptomatic testing and individuals with severe symptoms who sought tertiary care at the facility); or sampling and sequencing a portion of positive specimens collected at (3) 10%, (4) 25%, (5) 50% and (6) 100% of all tertiary sentinel facilities.

## Results

### Performance of current guidance

**Fig. 2:**
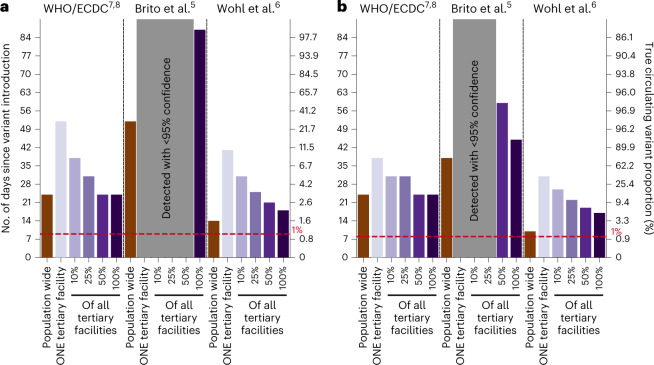
Performance of current guidance on number of positive specimens to sequence for variant detection with testing rate at 27 tests per 100,000 persons per day. First day of detection since variant introduction at 95% confidence and the corresponding circulating variant proportion using guidance from the World Health Organization (WHO)/European Centre for Disease Prevention and Control (ECDC)^7,8^, Brito et al.^5^ and Wohl et al.^6^ (Table 1) under different genomic surveillance strategies with varying sampling coverage (that is, all collected specimens from all healthcare facilities are sent to one facility to be sampled for sequencing (population-wide strategy); or only one tertiary facility or 10, 25, 50 or 100% of tertiary sentinel facilities would sample the specimens they collected for sequencing). Turnaround time (that is, time from specimen collection to acquisition of sequencing data) was assumed to be negligible. We performed 1,000 random independent simulations for each guidance/surveillance strategy. **a**,**b**, We simulated epidemics for wild-type SARS-CoV-2/Alpha (**a**) and Delta/Omicron (**b**). Gray regions denote that we could not reliably detect the variant virus with 95% confidence using the guidance in question under the assumed genomic surveillance strategy.

We first assessed various suggested sample sizes of positive specimens to sequence for detection of SARS-CoV-2 variants at low prevalence for simulated wild-type/Alpha and Delta/Omicron epidemics in Zambia with a mean testing rate of 27 tests per 100,000 people per day (based on the observed mean rate of testing in LMICs) (Fig. 2). We used recommended sample sizes from three prominent guidances: (1) The World Health Organization and European Centre for Disease Prevention and Control computed sample size using the binomial method^7,8^; (2) by subsampling genomic surveillance data generated in Denmark in 2020–2021 when the country was testing at more than 2,000 tests per 100,000 people per day on average, Brito et al.^5^ suggested that sequencing 0.5% of all detected cases, with a turnaround time of 21 days, would result in 20% variant detection before reaching 100 cases; and (3) Wohl et al.^6^ formulated a mathematical framework computing sequencing sample size by modeling the biological and logistical processes that impact sampled variant proportions. Critically, all three methods did not consider how low testing rates and spatial nonuniformity in sampling coverage impact sampled variant proportions and, in turn, speed of variant detection. The assumptions, mathematical background and lack of accounting for spatiotemporal bias in sample size estimation of each guidance are detailed in Table 1 and Supplementary Notes (see ‘Background on current guidance’ in Supplementary Notes).

**Table 1:** Current guidance by various stakeholder and academic groups on the number of specimens to sequence for detection of novel variants at low prevalence

	Recommendation on number/proportion of positive specimens to sequence	Critical considerations
World Health Organization/European Centre for Disease Prevention and Control^7,8^	Minimum number of sequences to detect at 1% variant proportion with 95% confidence for given number of reported cases: • 141 (<1,000 cases) • 196 (1,001–2,500 cases) • 243 (2,500–5,000 cases) • 270 (5,001–10,000 cases) • 285 (>10,000 cases)	• Agnostic to variant properties • Assumes specimen pool to be sampled for sequencing is representative of circulating diversity but acknowledges that, unless testing coverage is evenly distributed, this will be a biased sample • Notes that, in countries with limited sequencing capacity, monitoring relative prevalence of variants should be prioritized
Brito et al.^5^	At least 0.5% of all cases, with a turnaround time of 21 days to detect novel lineage before it reaches 100 cases at 20% probability	Based on sequencing data from Denmark, which is testing at an average of >2,000 tests per 100,000 persons per day^9^
Wohl et al.^6^	1–29 sequences per day to detect an Alpha-like variant based on 0.03% initial introduction for a population of 10,000 (assuming growth rate of 0.1 per day) at 1% variant proportion with 95% confidence^a^	• Assumes that the observed variant proportion in the positive specimens collected is representative of the circulating variant proportions among the infected population. This requires a large number of specimens that are randomly collected for assumption to hold true at low circulating variant proportions • A correction factor is included to correct for biases in the observed variant proportion, but only pertaining to those arising from the relative differences in diagnostic sensitivity, sample qualities and conditional asymptomatic and symptomatic testing probabilities between the two circulating variants

^a^We used the spreadsheet (https://github.com/HopkinsIDD/VOCsamplesize) provided and input appropriate parameters to obtain the recommendation relevant to the simulated epidemics.

As such, even when assuming negligible turnaround time (that is, time from specimen collection to acquisition of sequencing data), the recommended approaches were insufficient to detect the variant at their respective detection targets (for example, at 1% variant proportion or before 100 detected variant cases) when testing rates were low due to poor representativeness, regardless of the genomic surveillance sampling strategy. The first strategy of sampling specimens collected from the whole population that were sent to one sequencing facility (that is, population-wide strategy) led to the best performance (closest to detection target) for all recommendations because it involves random uniform sampling of all available samples, a fundamental assumption made by all current guidance. However, if the specimen pools available for sequencing are restricted to those collected from a subset of sentinel tertiary facilities only, the nonuniformity in sampling coverage results in spatiotemporal bias within the sequenced samples and leads to delayed detection of variants of concern (VOCs), which gets progressively worse as the proportion of tertiary facilities performing sequencing decreases to one facility.

### Variant detection

**Fig. 3:**
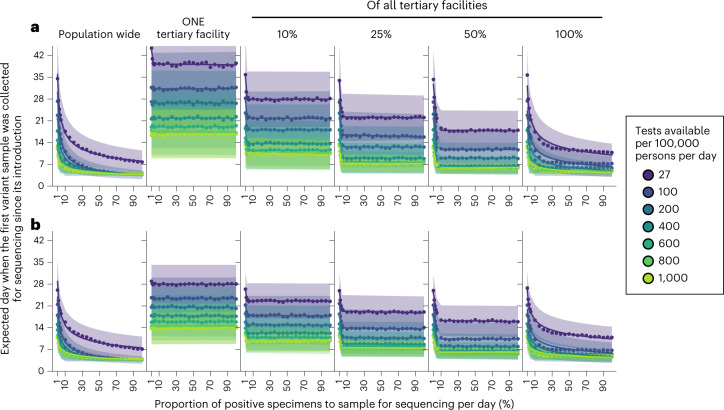
Impact of SARS-CoV-2 testing rates and proportion of positive specimens to sequence on variant detection. For each mean daily test availability (differently colored), the expected day (points and line) and the s.d. (shaded region) when the first variant specimen to be sequenced is sampled since its introduction is plotted against the proportion of positive specimens to be sampled for sequencing daily. Different genomic surveillance strategies with varying sampling coverage (that is, all specimens collected from all healthcare facilities sent to one facility to be sampled for sequencing (population-wide strategy); or only one tertiary facility, or 10, 25, 50 or 100% of tertiary sentinel facilities would sample the specimens they collected for sequencing) were simulated. **a**, Wild-type SARS-CoV-2/Alpha. **b**, Delta/Omicron. The plotted results were computed from 1,000 random independent simulations for each surveillance strategy.

To elucidate how SARS-CoV-2 testing rates and the proportion of positive specimens sequenced impact the speed of variant detection, we simulated wild-type SARS-CoV-2/Alpha and Delta/Omicron epidemics at different Ag-RDT availability, ranging from 27 to 1,000 tests per 100,000 persons per day (Fig. 3). We assumed that specimens to be sequenced are sampled on their collection day, and varied the proportion of positive specimens to sample for sequencing each day between 1 and 100%. We analyzed the impact of testing rates and sequencing proportions on the expected day when the first specimen sampled for sequencing containing the variant was collected as a measure of variant detection speed. In Fig. 3, we did not consider the time between sample collection and sequencing nor the turnaround time to obtaining sequencing results as they would only delay the actual day of variant detection by the assumed turnaround time.

For all testing rates, the relationship between the expected day when the first sample containing the variant was collected and the proportion of positive specimens sequenced per day can be described by a convex operating curve, reflecting rapidly diminishing returns in the speed of variant detection as more specimens are sampled for sequencing. Across all genomic surveillance sampling strategies, relatively larger marginal improvements to the speed of variant detection are generally made when the sequencing proportion is increased up to approximately 10% of all samples collected. Further sequencing only minimally shortens the expected time to variant detection, as the operating curve asymptotically approaches the earliest possible day of detection. Importantly, increasing SARS-CoV-2 testing allows smaller sequencing proportions to attain similar detection day targets, and higher testing rates lower the earliest possible detection day. For both the Alpha and Omicron variants, increasing testing rates from 27 to 100 tests per 100,000 persons per day brings forward the expected day of sampling the first variant sequence by at least 1 week (Fig. 3).

For the same level of testing and sequencing proportion, the population-wide strategy led to the earliest initial detection of a variant sequence. If sequencing were restricted to samples collected at a subset of tertiary sentinel facilities only, increasing the number of facilities sending samples for sequencing reduced the spatiotemporal bias in the specimen pool, thereby shaping the operating curves closer to the ones observed for the population-wide strategy. Interestingly, results similar to the population-wide strategy could be attained if all tertiary facilities acted as sentinel sites and sent the samples they collected for sequencing to increase the representativeness of sampling.

### Observed variant proportion

**Fig. 4:**
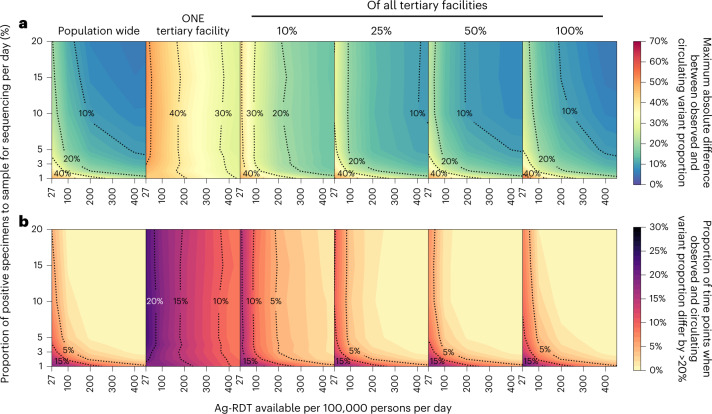
Impact of SARS-CoV-2 testing rates on the capacity to monitor changes in variant prevalence based on diagnostic test availability and proportion of test-positive samples sequenced. Different genomic surveillance strategies (that is, all specimens collected from all healthcare facilities sent to one facility to be sampled for sequencing (population-wide strategy); or only one tertiary facility or 10, 25, 50 or 100% of tertiary sentinel facilities would sample the specimens they collected for sequencing) were simulated. **a**, Maximum absolute difference between observed and circulating variant proportions. **b**, Proportion of time points when sequencing was performed that the absolute difference between observed and circulating variant proportions is greater than 20%. All results were computed from 1,000 random independent simulations for each surveillance strategy.

**Extended Data Fig. 1:**
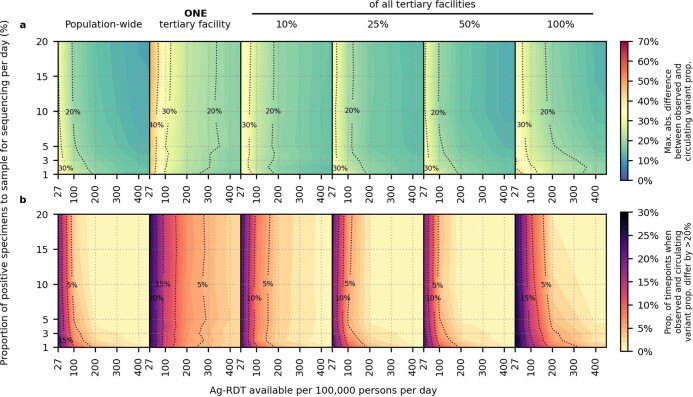
Impact of SARS-CoV-2 Ag-RDT testing rates and daily proportion of positive specimens to sample for sequencing on observed Omicron variant proportions. Different genomic surveillance strategies (that is all specimens collected from all healthcare facilities sent to one facility to be sampled for sequencing (*population-wide* strategy); only *one*, 10%, 25%, 50% or 100% of all tertiary facilities acting as sentinel sites that would sample the specimens they collected for sequencing) were simulated. (**A**) Maximum absolute difference between observed and circulating variant proportions. (**B**) Proportion of timepoints when sequencing was performed that the absolute difference between observed and circulating variant proportions is greater than 20%. All results were computed from 1,000 random independent simulations for each surveillance strategy.

Test availability and sampling coverage also affect the accuracy of the observed variant proportion (Fig. 4 and Extended Data Fig. 1). At a testing rate of 27 tests per 100,000 persons per day, the observed variant proportion maximally differs from the true circulating proportion by more than 30% of the true value for both the Alpha and Omicron variants and, for more than 15% of the time, the proportional difference between the observed and true variation was greater than 20%. Both the maximum absolute difference and percentage of time points where the difference is greater than 20% can be lowered to less than 20% and less than 5%, respectively, if the testing rate is increased to 100 or more tests per 100,000 people per day.

Critically, when the representativeness of the specimen pool is spatiotemporally biased by sequencing samples collected at tertiary sentinel facilities only, increasing the proportion of specimens to be sequenced only marginally lowers the maximum absolute difference or lessens the number of times where observed variant proportion deviates less than 20% from true circulating proportions (Fig. 4, near vertical isoclines at low daily rates of testing). Increasing testing rates at sentinel surveillance sites provides more accurate detection in changes to circulating prevalence than sequencing more samples in the context of low testing rates.

### Sensitivity analyses

We repeated our analyses using virus properties (that is, incubation period, maximum viral load, protection against infection by the mutant virus after extant virus infection) of the Omicron variant, but varied different relative transmissibility to the Delta variant (1.0 to 4.0) and the initial proportion of individuals who had been infected by the Delta variant (10 and 40%). The variant growth rates simulated for these hypothetical Delta/Omicron epidemics ranged from 0.17 to 0.42 per day.

**Extended Data Fig. 2:**
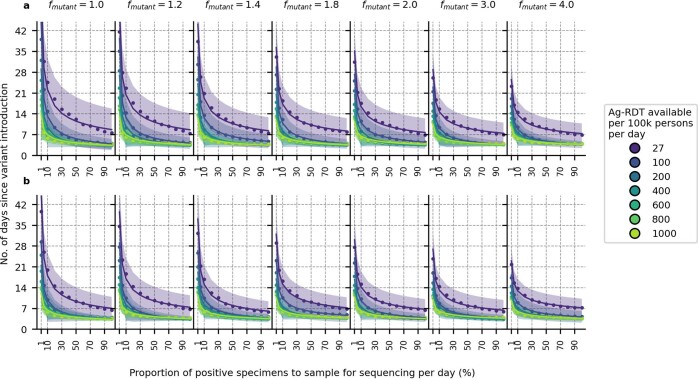
Sensitivity analyses on variant detection operating curve for different relative transmissibility factor. For each Ag-RDT availability (differently colored), the expected day (points and line) and the standard deviation (shaded region) when the first Omicron variant specimen (in the background of extant Delta variant) is sampled for sequencing since its introduction is plotted against the proportion of positive specimens to be sampled for sequencing daily. All specimens collected from the population from all healthcare facilities were sent to one facility to be sampled for sequencing (population-wide genomic surveillance strategy). Different transmissibility factor of Omicron relative to Delta (*f*_*mutant*_) were assumed. (**A**) 10% and (**B**) 40% of the population had immunity against Omicron initially. The plotted results were computed from 1,000 random independent simulations for each surveillance strategy.

**Extended Data Fig. 3:**
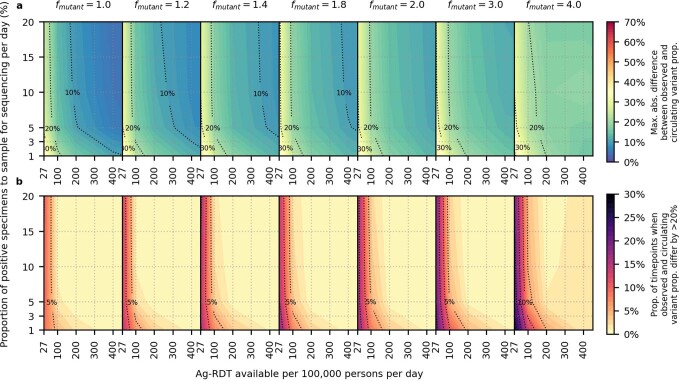
Sensitivity analyses on accuracy of observed variant proportions for different relative transmissibility factor. Omicron-like virus properties assumed for variant and initial proportion of population with some degree of protection against the variant virus assumed at 10%. All specimens collected from the population from all healthcare facilities were sent to one facility to be sampled for sequencing (population-wide genomic surveillance strategy). Different transmissibility factor of Omicron relative to Delta (*f*_*mutant*_) were assumed. (**A**) Maximum absolute difference between observed and circulating variant proportions. (**B**) Proportion of timepoints when sequencing was performed that the absolute difference between observed and circulating variant proportions is greater than 20%. All results were computed from 1,000 random independent simulations for each surveillance strategy.

**Extended Data Fig. 4:**
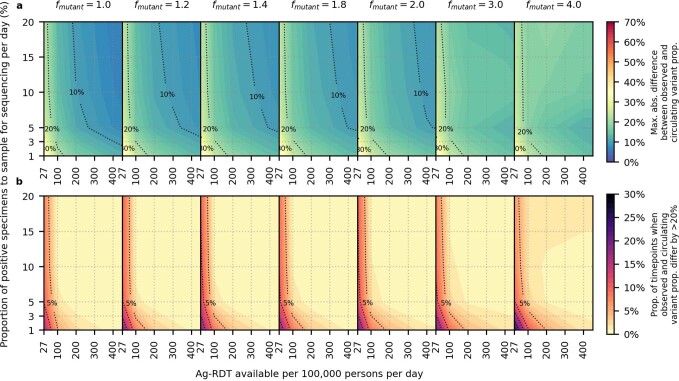
Sensitivity analyses on accuracy of observed variant proportions for different relative transmissibility factor. Omicron-like virus properties assumed for variant and initial proportion of population with some degree of protection against the variant virus assumed at 40%. All specimens collected from the population from all healthcare facilities were sent to one facility to be sampled for sequencing (population-wide genomic surveillance strategy). Different transmissibility factor of Omicron relative to Delta (*f*_*mutant*_) were assumed. (**A**) Maximum absolute difference between observed and circulating variant proportions. (**B**) Proportion of timepoints when sequencing was performed that the absolute difference between observed and circulating variant proportions is greater than 20%. All results were computed from 1,000 random independent simulations for each surveillance strategy.

Under these varied conditions, the expected day when the specimen of the first variant sequence is collected still follows a convex-shaped operating curve against the daily proportion of positive specimens to sequence. For all curves, the larger marginal improvements in shortening variant detection are still in sequencing proportions of up to approximately 10% (Extended Data Fig. 2). In terms of the accuracy of observed variant to true circulating proportions, the maximum absolute difference and percentage of time points where the difference is greater than 20% are both substantially lowered if the testing rate is increased to at least 100 tests per 100,000 people per day (Extended Data Figs. 3 and 4).

**Extended Data Fig. 5:**
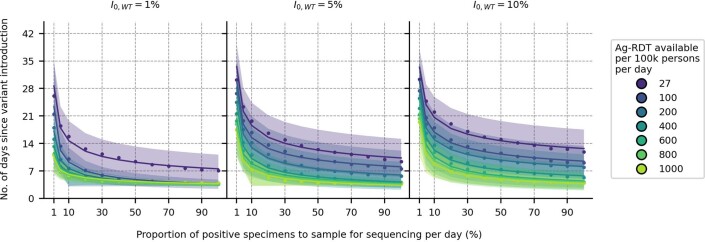
Impact of prevalence of extant variant of concern (*I*_0*,WT*_) at the time of new variant introduction. For each Ag-RDT availability (differently colored), the expected day (points and line) and the standard deviation (shaded region) when the first Omicron variant specimen (in the background of Delta) is sampled for sequencing since its introduction is plotted against the proportion of positive specimens to be sampled for sequencing daily. Each panel shows a different prevalence of the Delta variant (*I*_0*,WT*_) at the point of Omicron introduction. Sampling for sequencing was drawn from the population-wide scenario. The plotted results were computed from 1,000 random independent simulations for each surveillance strategy.

We also varied the prevalence of extant Delta infections when the Omicron variant was introduced (Extended Data Fig. 5). We found that lower test availability causes a delay in sampling the first variant specimen if the variant is introduced when pre-existing extant variant circulation is high. At 27 tests per 100,000 persons per day, regardless of specimen proportions sequenced, detection could be delayed by approximately 1 week if Omicron was introduced when Delta was circulating at 10% prevalence as opposed to 1%. This is because a greater share of tests would be used to diagnose the more prevalent extant virus infections which, in turn, decreases the likelihood of detecting the newly introduced variant at low proportions.

## Discussion

Our findings show that the emphasis on the proportion of samples referred for genomic surveillance is misplaced if testing capacity is insufficient and sample sources are highly spatiotemporally biased. As such, at the current mean rate of testing in LMICs (27 tests per 100,000 persons per day), current guidance^5–8^ on sequencing sample size estimation could likely lead to later-than-predicted detection of novel variants at best or, at worst, leave new variants undetected until they have infected a majority of a population.

**Fig. 5:**
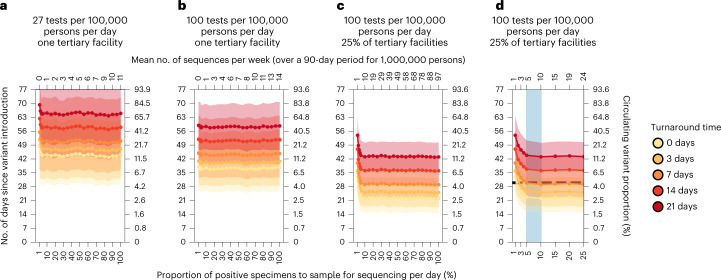
Recommended approach to enhance genomic surveillance robustness. In each plot, the operating curves of the expected day when the first Alpha variant sequence is generated are plotted for different proportions of specimens to sample for sequencing per day and turnaround times. We assumed that the Alpha variant was circulating at 1% initially, with wild-type SARS-CoV-2 in the background. We also assumed that positive specimens sampled within each week for sequencing are consolidated into a batch before they are referred for sequencing. Turnaround time refers to the time between collection of each weekly consolidated batch of positive specimens to the acquisition of its corresponding sequencing data. The vertical axes denote the number of days passed since the introduction of the Alpha variant (left) and its corresponding circulating proportion (right). The horizontal axes denote the proportion of positive specimens to sample for sequencing per day (bottom) and the corresponding mean number of sequences to be generated per week per 1,000,000 people over a 90-day epidemic period. **a**, Specimen pools for sequencing from *one* tertiary sentinel facility with testing rate at 27 tests per 100,000 persons per day. **b**, Specimen pools for sequencing from *one* tertiary sentinel facility with testing rate at 100 tests per 100,000 persons per day. **c**, Specimen pools for sequencing from 25% of all tertiary sentinel facilities with testing rate at 100 tests per 100,000 per day. **d**, Zoomed-in plot of **c** to highlight sequencing proportions varying between 1 and 25%. Sequencing 5–10% of positive specimens (blue shaded region) would ensure that we would expectedly detect Alpha in 30 days (horizontal dashed line) if turnaround time is kept within 1 week. All results were computed from 1,000 random independent simulations for each surveillance strategy. The shaded region depicts the s.d. across simulations.

Based on our work, we identified three major areas of improvement that could be prioritized to enhance the robustness of genomic surveillance programs (Fig. 5). First, the most substantial improvements are likely to come from increasing the mean testing rate in LMICs from 27 tests per 100,000 persons per day (Fig. 5a) to at least 100 tests per 100,000 persons per day (Fig. 5b). Even if sentinel surveillance was conducted at only one tertiary facility, this increase in testing rate for the catchment area of the facility would likely speed up variant detection by 1–2 weeks.

Second, the representativeness of a specimen pool for sequencing can be further improved by expanding sampling coverage. In our model, variant detection was further sped up by 1–3 weeks by increasing the percentage of tertiary sentinel facilities sending the samples they had collected for sequencing to 25% of facilities (Fig. 5c). Additionally, in terms of prevalence monitoring, if 25% of tertiary facilities sequenced 5% of all positive specimens they had collected to detect and monitor an Alpha-like variant, the maximum absolute difference to the true circulating proportion is expected to decrease from more than 50% (assuming a single sentinel facility) to no more than 20%.

Third, reducing turnaround time from samples referred to sequencing output results in a 1:1 decrease in time to new variant detection, regardless of the proportion of sequenced samples, test availability or sampling coverage (Fig. 5). These gains require scaling up in sample transport networks, access to sequencing machinery, trained personnel and/or increases in numbers of sequenced samples to make the most efficient use of each sequencing run^11^. Furthermore, LMICs also often face high costs and extended delivery delays of laboratory reagents and consumables that were sometimes further exacerbated by recurring travel bans during the acute phase of the pandemic^5,12,13^.

**Extended Data Fig. 6:**
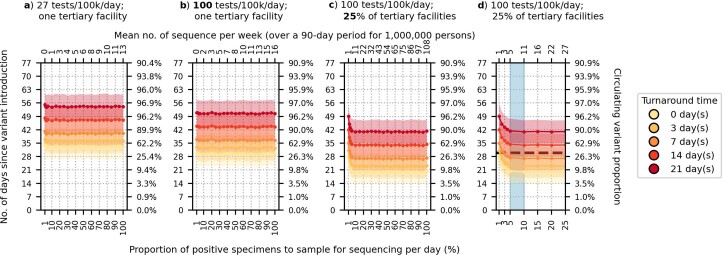
Recommended approach to enhance genomic surveillance robustness. In each plot, the operating curves of the expected day when the first Omicron BA.1 variant sequence is generated are plotted for different proportion of specimens to sample for sequencing per day and turnaround times. We assumed that the Omicron BA.1 variant was circulating at 1% initially with Delta variant in the background. We also assumed that positive specimens sampled within each week for sequencing are consolidated into a batch before they are referred for sequencing. Turnaround time refers to the time between collection of each weekly consolidated batch of positive specimens to the acquisition of its corresponding sequencing data. The vertical axes denote the number of days passed since the introduction of the Omicron variant (left) and its corresponding circulating proportion (right). The horizontal axes denote the proportion of positive specimens to sample for sequencing per day (bottom) and the corresponding mean number of sequences to be generated per week per 1,000,000 people over a 90-day epidemic period. (**A**) Specimen pools for sequencing from *one* tertiary facility with testing rate at 27 tests per 100,000 persons per day (tests/100 k/day). (**B**) Specimen pools for sequencing from *one* tertiary sentinel facility with testing rate at 100 tests/100 k/day. (**C**) Specimen pools for sequencing from 25% of all tertiary facilities acting as sentinel sites with testing rate at 100 tests/100 k/day. (**D**) Zoomed-in plot of (**C**) for sequencing proportions varying between 1–25%. Sequencing 5–10% of positive specimens (blue shaded region) would ensure that we would expectedly detect Omicron within 30 days if turnaround time is kept within one week. All results were computed from 1,000 random independent simulations for each surveillance strategy. The shaded region depicts the standard deviation across simulations.

After reducing spatiotemporal bias in the specimen pool through increased testing and sampling coverage, sequencing up to 5–10% of the positive specimens collected could return the greatest information gains while minimizing resource wastage. For an Alpha-like variant, 100 tests per 100,000 persons per day with sampling from 25% of tertiary sentinel facilities for sequencing amounts to an estimated 5–10 sequences per week averaged over a 90-day period per 1,000,000 people. If turnaround time is kept within 1 week, the variant would likely be detected within 1 month at a circulating proportion of approximately 4% (Fig. 5d). Similarly, at the same testing rate, sampling coverage and turnaround time (that is, average 5–11 sequences per week per 1,000,000 people), an Omicron-like variant would be detected before the first month since its introduction, but at approximately 23% circulating proportion owing to its faster transmission (Extended Data Fig. 6).

Our findings serve to inform expectations of genomic surveillance initiatives and should be interpreted according to the public health objectives of each program. If the objective is to serve as an early warning system for the de novo emergence of new variants before they are likely to have spread widely, then all factors above can be considered essential and could require substantially more than 100 tests per 100,000 persons per day. Critically, determining that a new variant is a threat requires not only detection of the variant itself but also the capacity to reliably monitor changes in its prevalence and potential clinical impact on short timescales. The results presented here also inform the design of programs for the sensitive and reliable detection of changes in variant prevalence. Otherwise, if the objective is to detect for the introduction of novel variants from overseas, some of the factors above may be relaxed depending on the public health objectives. For instance, if the aim is to attempt containment, all factors should still be considered to promptly detect and monitor the spread of the variant. However, if the aim is to ensure sufficient time for control strategies to be enacted, less samples could be sequenced or turnaround time could be longer, for example, so long as the mitigation strategies remain useful when implemented.

Despite performing our simulations using demographic parameters from Zambia, the emergence and detection of each VOC to date represents interesting case studies for the work described here (see ‘Emergence of SARS-CoV-2 variants of concern’ in Supplementary Notes). For example, at the time of the first detection of the Omicron variant, in South Africa in November 2021, the daily SARS-CoV-2 testing rate was 51 tests per 100,000 people per day^9^, which was among the highest testing rates in Africa. However, the Omicron variant was only detected 6–8 weeks after its likely emergence^4^. At that point, Omicron had already infected a substantial portion of the population in Gauteng, South Africa (that is, the estimated circulating variant proportion was greater than 80% by mid-November)^4^. Not only had the variant already spread across the rest of South Africa and to neighboring Botswana^4^, Omicron samples were also collected in multiple other countries, including Hong Kong^14^, Denmark^15^ and the Netherlands^16^, before the initial reports on the identification of the Omicron variant. This situation is consistent with our modeling findings, where novel variant detection is possible with less than 100 tests per 100,000 persons per day, but only after the new variant has spread widely across the population.

In another example, Germany randomly sequenced approximately 60–70 sequences per week (that is, less than 1% of cases sequenced per day) in December 2020^17^. During this time, testing rates in Germany averaged at approximately 300 tests per 100,000 persons per day^9^. Germany was able to detect the Alpha variant 1 week before the World Health Organization declared the lineage a VOC in mid-December 2020^17^. The Alpha variant likely emerged in the UK in mid-September 2020^18^ and rapidly proliferated across the country before it was reported in December 2020^19^. Our analyses showed that the expected time before the first Alpha variant specimen was sampled for sequencing since its introduction is more than 4 weeks (that is, around November 2020) at Germany’s testing and sequencing rate. This falls in line with the likely period of Alpha’s introduction into Germany, similar to the period estimated for its European neighbors, such as the Netherlands^20^.

**Extended Data Fig. 7:**
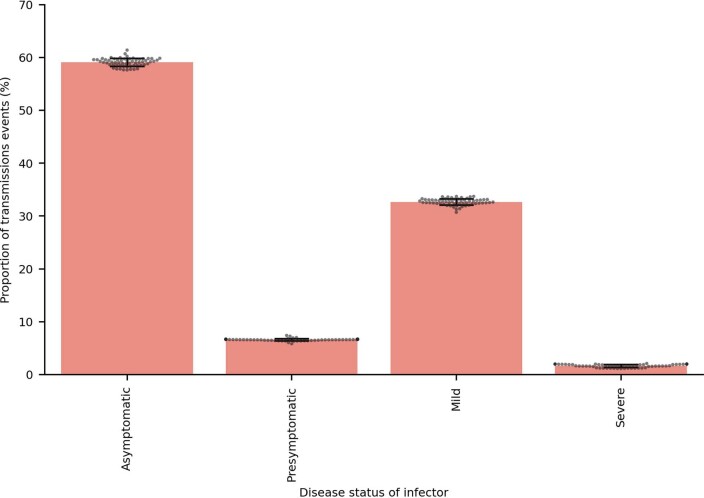
Transmissions attributed to infectors of different disease status. Proportion of transmissions events (data points) attributed to different disease status of infectors across all independent epidemic simulations (n = 280). Bar plots show the mean proportion with error bars denoting ± standard deviation.

**Extended Data Fig. 8:**
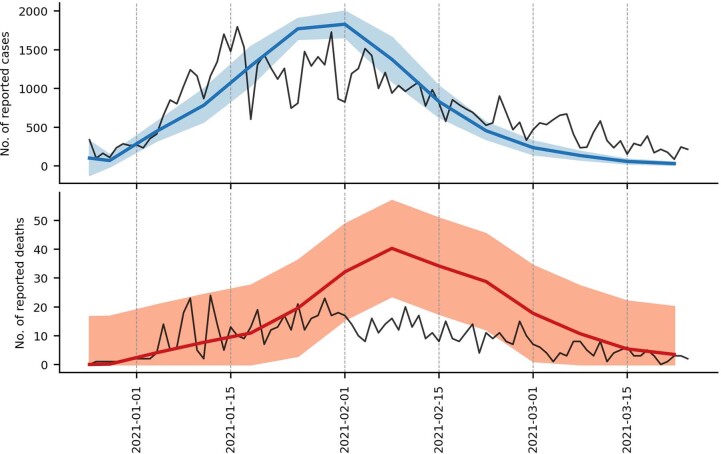
Model validation. We compared the mean number of reported cases (blue line, top panel) and deaths (red line, bottom panel) estimated by our simulations (10 simulations in total; see Supplementary Text) against the actual case and death counts (black lines) in Lusaka, Zambia during the second wave of infections between 25 December 2020 and 24 March 2021. Actual case and death counts were retrieved from the Zambia COVID-19 Dashboard (https://www.arcgis.com/apps/dashboards/3b3a01c1d8444932ba075fb44b119b63). The blue and red shaded regions in each plot denotes the standard deviation of reported cases (top panel) and deaths (bottom panel) respectively.

There are some limitations to our work. First, although we computed the amount of testing and sampling coverage required to achieve prompt and precise variant surveillance outcomes, comprehensive cost-effectiveness analyses are needed to determine the cost-optimal approach toward expanding testing programs that support surveillance alongside other epidemic control objectives. Second, PATAT iteratively simulates the course of an epidemic wave in time steps of 1 day. As a simplification, PATAT assumes a logical flow where testing and isolation after positive diagnoses occur before transmissions are simulated each day. However, in reality, transmission could occur before testing and isolation and thus potentially lead to an underestimation in infections. Nonetheless, a substantial portion of SARS-CoV-2 transmissions are attributable to individuals who are asymptomatic and presymptomatic^21^, who would not seek testing until they present symptoms. This was reflected in our simulations (Extended Data Fig. 7). As such, it is unlikely our simulations substantially underestimate disease spread. Furthermore, other agent-based SARS-CoV-2 transmission models that made similar assumptions were validated against real-world epidemiological data^22,23^. Importantly, our simulation results also fit well against confirmed case and death counts in Zambia when accounting for the prevailing testing rates in the country (see ‘Model validation’ in Supplementary Notes and Extended Data Fig. 8).

Although we find that routine representative sampling is vital for monitoring SARS-CoV-2 evolution, additional surveillance systems, including targeted surveillance of particular populations and settings (such as individuals who are immunocompromised or unusual events) and wastewater sampling, could enable increased variant detection sensitivity^24^. In particular, recent advances in wastewater sequencing and deconvolution methods to resolve multiple viral lineages in mixed wastewater samples enabled detection of emerging variants before they were captured by clinical genomic surveillance^25–27^. However, sequence quality is often poor in wastewater samples and, in turn, these methods depend on a priori knowledge of the lineage-defining mutations of VOCs and variants of interest, which are currently still identified based on noteable upsurges in individuals with clinical diagnoses. Furthermore, centralized wastewater management systems, which these methods rely on for accurate determination of relative lineage prevalence, are currently nonexistent in many LMICs. Substantial investments, coordination and time are needed to enable local sanitation infrastructures suitable for wastewater surveillance^28^. Detection of genetic markers, such as *S*-gene target failure in PCR assays, may also provide faster notification of viral lineages with these specific mutations. However, whole-genome sequencing is still needed for unambiguous genotyping of SARS-CoV-2 samples. Ultimately, clinical diagnostic testing and surveillance will remain the core mode of SARS-CoV-2 surveillance in most countries.

During the initial phase of the pandemic in 2020, due to limited testing and sequencing capacities, many LMICs were initially focused on genomic surveillance efforts at points of entry at country borders to deter introductions^29–31^. Over time, especially after the emergence of VOCs, SARS-CoV-2 genomic surveillance gradually expanded to include community surveillance as many LMICs enhanced their sequencing capacities^4,31–33^. This was done either by establishing regional sequencing networks to maximize available resources, investing in local sequencing capacities or partnering with global collaborators^33–35^. Sequencing turnaround time has also improved from an average of approximately 170 days in 2020 to approximately 30 days in 2021 across the African continent, albeit with substantial variation among countries^33^. Although sequencing capabilities have expanded in LMICs, obtaining spatiotemporally representative samples remains a key challenge^33^. Our work shows that the sensitivity of genomic surveillance programs is highly dependent on diagnostic testing rate and that a mean testing rate of 100 tests per 100,000 persons per day at sentinel sites that are geographically spread out across the community is a good basis for monitoring virus variants. Whereas a reflexive PCR test after a positive Ag-RDT diagnosis is currently performed to obtain samples suitable for sequencing (and is possible in many tertiary facilities in LMICs), this presents additional cost and logistical barriers. Recent studies showed that SARS-CoV-2 sequencing can be performed using materials obtained from Ag-RDTs performed at point of care^36–38^. Importantly, whole genomes can be recovered up to 8 days after testing, providing opportunities for sequencing to be performed on samples performed through self-testing as well^36^.

Expanding genomic sequencing capabilities, especially in LMICs, is a global priority^39^ and current investments in sequencing must continue^33,34^. Simultaneously, sustained investments in public health systems are required to expand access to, and availability of, diagnostic testing to underpin SARS-CoV-2 surveillance programs. Here, we primarily focused on LMICs, but our findings on the impact of testing rates and representativeness on genomic surveillance programs are equally important for HICs as parts of their testing and surveillance infrastructures are dismantled following the acute health emergency of the COVID-19 pandemic. Ultimately, detecting the next SARS-CoV-2 variant or pathogen that causes the next pandemic requires fundamental clinical diagnostic capacity to monitor existing and emerging pathogens.

## Methods

### Simulating SARS-CoV-2 epidemics with the PATAT model

We used PATAT, a stochastic, individual-based model to simulate SARS-CoV-2 epidemics in a community with demographic profiles, contact mixing patterns and level of public health resources mirroring those typically observed in LMICs. Here, the model was based on Zambia. PATAT creates an age-structured population, linking individuals within contact networks of multigenerational households, schools, workplaces and churches (that is, regular mass gatherings) (Supplementary Table 1). The simulated number of healthcare facilities (that is, community clinics and tertiary hospitals) where individuals with mild symptoms seek symptomatic testing and have their virus specimens collected was based on an empirical clinic to population ratio (that is, one healthcare facility for every 7,000 individuals, on average)^40,41^. Although PATAT does not explicitly simulate the spatial location of individuals, contact networks and healthcare facilities are ordered to approximate localized community structures (that is, the closer the number order of a facility, the closer they are in the same neighborhood) that is most illustrative of urban centers. Households are proximally ordered and distributed around these facilities based on an empirical distance-structured distribution that correlates with probabilities of individuals with symptoms seeking testing at clinics (Supplementary Table 1).

We then simulated SARS-CoV-2 infection waves in a population of 1,000,000 individuals over a 90-day period that begins with an initial 1% prevalence of an extant SARS-CoV-2 variant and the introduction of a mutant variant at 0.01%. We assumed that clinic-based, professional-use Ag-RDTs are the predominant SARS-CoV-2 diagnostic used for SARS-CoV-2 testing^39^. Because Ag-RDT sensitivity depends on within-host viral loads^42^, PATAT generates viral load trajectories, measured in cycle threshold values, for individuals with COVID-19 by randomly sampling from known viral load distributions of different SARS-CoV-2 variants^43,44^. We performed simulations for two variant replacement scenarios, Alpha variant introduction while the wild-type virus was circulating (wild-type/Alpha) and Omicron (BA.1) variant introduction while Delta was circulating (Delta/Omicron), applying known distributions of their peak viral load, incubation and virus clearance periods^43,45^ (Supplementary Table 1). Before simulating the two-variant epidemic, we first calibrated the transmission probability parameter for the extant variant such that it would spread in a completely susceptible population at *R*_0_ = 2.5–3.0. We then assumed Alpha and Omicron (BA.1) were more transmissible than the respective extant virus to achieve growth rates of approximately 0.15 and 0.35 per day, respectively^2,10^.

For both sets of simulations, we assumed that 10% of the population had infection-acquired immunity against the extant strain initially, with some level of protection against infection by the mutant virus (wild-type SARS-CoV-2, 80% protection against Alpha^46^; Delta, 20% protection against Omicron^10^). We also investigated the scenario where 40% of the population had infection-acquired immunity as part of sensitivity analyses (see below). We did not investigate scenarios involving vaccine-acquired immunity due to low vaccine uptake in most LMICs^47^.

PATAT uses the SEIRD (susceptible-exposed-infected-recovered/death) epidemic model for disease progression and stratifies individuals who are infected on the basis of their symptom presentation (asymptomatic, mild or severe). After an assumed random delay after symptom onset (mean = 1 day; s.d. = 0.5 day), individuals with symptoms who seek testing would do so at their nearest healthcare facility, where test-positive samples may be reflexively collected for sequencing. We assumed that individuals with symptoms sought testing based on a probability distribution of health service-seeking behavior that inversely correlates with the distance between the individual’s household and the nearest healthcare facility (Supplementary Table 1)^48^.

We varied levels of Ag-RDT stocks per day (that is, 27, 100 and 200–1,000 (in increments of 200) tests per 100,000 persons per day), running ten independent epidemic simulations for each testing rate. Given the start of a week on a Monday, we assumed that a week’s worth of tests are delivered to healthcare facilities every Monday and unused Ag-RDTs in the previous week are carried forward into the next week. If test stocks for a particular week were exhausted before the end of the week, testing for the rest of that week ceased. Due to overlapping symptoms between COVID-19 and other respiratory diseases, a proportion of available Ag-RDTs would be used by individuals who are not infected with SARS-CoV-2. Based on test positivity rates reported by various countries in the second half of 2021^49^, we assumed a 10% test positivity rate at the start and end of the simulated epidemic, and 20% test positivity at its peak, linearly interpolating the rates between these time points. We also assumed that false-positive specimens could be sampled based on a reported Ag-RDT specificity of 98.9%^42^.

We assumed that any specimens collected for genomic surveillance after positive detection through Ag-RDT would be reflexively confirmed with PCR. We also assumed that all individuals who were symptomatic with severe symptoms require hospitalization, and are tested separately from persons with mild symptoms who sought testing. Given that likely only approximately 10–20% of people who died from COVID-19 in Zambia were tested for the disease in life^50,51^, we assumed that only 20% of individuals with severe disease would be tested by Ag-RDT or PCR upon presenting severe symptoms and have specimens collected for sequencing.

Full technical details of PATAT are described in the Supplementary Information. The full model source code is available at https://github.com/AMC-LAEB/PATAT-sim.

### Genomic surveillance strategies

Twenty percent of healthcare facilities were assumed to be tertiary facilities based on empirical data collected from Zambia^40,41^. We assumed that tertiary facilities provide testing for individuals with mild symptoms and hospitalized patients with severe symptoms. Given that healthcare facilities were proximally ordered, we randomly selected tertiary facilities in each independent surveillance simulation (see below), but ensured that the selected facilities were not consecutively ordered. In sum, all tertiary facilities accounted for a median of 18.4% (interquartile range = 17.7–19.1%) of total testing volume across all simulations. We assumed that a proportion of tertiary facilities serve as sentinel surveillance sites that reflexively collect SARS-CoV-2-positive samples for sequencing. We then simulated six strategies with varying degrees of sampling coverage, where positive specimens collected from testing sites would be consolidated and sampled for sequencing: (1) all samples from community clinics and tertiary hospitals are sent to a centralized facility and further sampled for sequencing (that is, population-wide strategy); (2) only one tertiary sentinel facility for the population of 1,000,000 simulated people would sequence a portion of positive specimens it has collected, both from individuals with mild symptoms seeking symptomatic testing and individuals with severe symptoms who sought tertiary care at the facility; or only (3) 10%, (4) 25%, (5) 50% and (6) 100% of all tertiary sentinel facilities would sample and sequence a proportion of the specimens they have collected.

For all strategies, we assumed that a proportion (1–100%; in 2% increments between 1 and 5%, in 5% increments between 5 and 100%) of positive specimens are collected daily for sequencing. We also assumed that positive specimens sampled within each week for sequencing are consolidated into a batch before they are referred for sequencing. Turnaround time refers to the time between collection of each weekly consolidated batch of positive specimens to the acquisition of its corresponding sequencing data. Because the within-host viral loads of individuals infected with SARS-CoV-2 were simulated, we assumed that only high-quality samples, where cycle threshold values less than 30, could be sequenced and that the sequencing success rate is 80%, as assumed in other studies^6^.

For each strategy and sequencing proportion, we performed 100 independent surveillance simulations for each epidemic simulation with a given test stock availability, thus totaling to 1,000 random simulations for each set of variables (that is, testing rate, sequencing proportion and strategy).

### Statistics and reproducibility

No statistical method was used to predetermine the population size in our agent-based modeling study. We chose to simulate a population size of 1,000,000 individuals because it is sufficiently large enough to generate the desired epidemic characteristics and inferences on surveillance outcomes. We validated our simulation results based on this population size against real-life reported case count data in Zambia (see ‘Model validation’ in Supplementary Notes). All simulation data generated were included in our analyses.

### Ethics statement

Ethics approval was not required for this study.

### Reporting summary

Further information on research design is available in the Nature Portfolio Reporting Summary linked to this article.

## Online content

Any methods, additional references, Nature Portfolio reporting summaries, source data, extended data, supplementary information, acknowledgements, peer review information; details of author contributions and competing interests; and statements of data and code availability are available at 10.1038/s41588-022-01267-w.

## Supplementary information

Supplementary Information Supplementary Notes and Table 1.

Reporting Summary

## Data Availability

Data on global testing rates were downloaded from https://www.finddx.org/covid-19/test-tracker. All data used to parameterize the PATAT simulation model can be found in the article and Supplementary Information. All simulation data generated for this study can be found in the GitHub repository (https://github.com/AMC-LAEB/PATAT-sim).

## Code availability

The PATAT model source code and custom codes used to analyze our simulation data are available at https://github.com/AMC-LAEB/PATAT-sim and https://github.com/AMC-LAEB/PATAT-sim/blob/main/projects/surveillance/han-et-al_genome_surveillance_lmics.ipynb, respectively. This version of the model and analysis codes is also available at 10.5281/zenodo.7308781.
