# Supplementary material for: SARS-CoV-2 diagnostic testing rates determine the sensitivity of genomic surveillance programs
Source: Nat Genet. 2023 Jan 9;55(1):26–33. doi: 10.1038/s41588-022-01267-w (PMC9839449; doi:10.1038/s41588-022-01267-w)
Supplement: Supplementary file 1 — Supplementary Notes and Table 1. [file 41588_2022_1267_MOESM1_ESM.pdf]

nature genetics

Article https://doi.org/10.1038/s41588-022-01267-w

SARS-CoV-2 diagnostic testing rates determine the sensitivity of genomic surveillance programs

In the format provided by the authors and unedited

Supplementary Information

SARS-CoV-2 diagnostic testing rates determine the sensitivity of genomic surveillance programs

Alvin X. Han[1,*], Amy Toporowski[2], Jilian A. Sacks[3], Mark D. Perkins[3], Sylvie Briand[3], Maria van Kerkhove[3], Emma Hannay[2], Sergio Carmona[2], Bill Rodriguez[2], Edyth Parker[4], Brooke E. Nichols[1,2,5,†], Colin A. Russell[1,5,†,*]

[1]Department of Medical Microbiology & Infection Prevention, Amsterdam University Medical Center, University of Amsterdam, Amsterdam, The Netherlands

[2]Foundation for Innovative New Diagnostics (FIND), Geneva, Switzerland

[3]Department of Epidemic and Pandemic Preparedness and Prevention, Emergency Preparedness Programme, World Health Organization, Geneva, Switzerland

[4]Department of Immunology and Microbiology, The Scripps Research Institute, La Jolla, CA, USA

[5]Department of Global Health, School of Public Health, Boston University, Boston, MA, USA

†Contributed equally

*Corresponding authors. Email: x.han@amsterdamumc.nl and c.a.russell@amsterdamumc.nl

Supplementary Notes

Technical details of the PATAT simulation model

The computational flow of a PATAT simulation is summarized as follows: First, an age-structured population of agents is created. Close contact networks are subsequently created based on the given demographic data. The simulation is then initialized and iterates over a given period of time where each time step corresponds to a day. The sequential operations during each timestep follow the following order: (1) update the disease progression of infected individuals, (2) update the status of isolated/quarantined agents, (3) application of community testing strategies and (4) computation of transmission events within contact networks.

Population demography

Using input demographic data which includes information such as population age and sex distribution, household composition, employment and schooling rates, PATAT generates a population of individuals who are linked by a series of underlying contact network settings where transmission may occur. These contact network settings include households, schools, workplaces, regular mass gatherings (i.e. church) as well as random community contacts.

Household

PATAT randomly generates a Poisson distribution of household sizes based on the given mean household size. A reference individual (e.g. head of the household) above an assumed prime adult age (e.g. 20 years) is first randomly assigned to each household. To account for multigenerational households, the remaining household members are then randomly sampled multinomially by the input age distribution of households. Although PATAT does not explicitly model the geolocation of agents, households are ordered to implicitly approximate neighbourhood proximity.

Schools

PATAT distinguishes between elementary and secondary schools. For each education level, schooling children are randomly sampled from the population based on given enrolment rates and gender parity. Class sizes are then randomly drawn from a Poisson distribution based on the input mean class size while constrained by the number of schooling children attending the same grade (i.e. age; a class include only students studying the same grade). Schools are created by random allotment of classes such that (i) all schools will have equitable distributions of classes of all grades for the given education level and (ii) the total number of students approximately equals to the expected school size. Classes are then populated by schooling agents such that (i) agents of proximally ordered households will tend to attend the same school and (ii) children of

the same grade (age) from identical households will not be assigned to the same class even though they may attend the same school. School teachers are then randomly drawn from the employed prime adult population based on the input teacher-to-student ratio and are assumed to have contact with each other during school days. Each class is randomly assigned to one teacher.

Workplaces
PATAT generates both formal and informal workplace contact networks based on separate employment rates. Youth (15-19 years) employment is also considered in the potential workforce. The distinction between formal and informal settings is made as mean employee contact rates likely differ between them. Furthermore, workplace distribution of Ag-RDTs for community testing is assumed to be feasible for formal employment entities only. Unlike schools, PATAT does not explicitly model for workplaces but sets up contact matrices between employed individuals who would be in regular contact at work. Different sizes of workplace contact networks are randomly drawn from a Poisson distribution based on the given mean employee contact size. An employed agent would only be associated with one workplace contact network.

Mass gatherings (Churches)
High-density mass gatherings are considered in the model in the form of contacts among church congregations given the large weekly worship attendance in Zambia (i.e. >70%)[1] which we had modelled as our prototypical low-income country. The size of a church is assumed to follow a truncated Normal distribution with the given mean and variance with size greater than one. All floating-point size draws are rounded to the nearest integer. PATAT assumes that all members of a household will visit a church together every Sunday. Other than close contacts with each other, each household member would also have a random number of close contacts from other households that attend the same church. This random contact number is drawn from a Gamma distribution with the given shape and scale parameters (Table S1) and must be greater than zero. Any floating-point number of contacts drawn are rounded to their nearest integer. Churches are also ordered such that proximally ordered households in the same neighbourhood would visit the same church.

Random community
PATAT assumes that every agent within a given age range would have a random number of contacts with the community daily, drawn from a Poisson distribution with a given mean.

Disease progression

PATAT implements a SEIRD epidemic model where the simulated population is distinguished between five compartments: susceptible, exposed (i.e. infected but is not infectious yet; latent phase), infected (which include the presymptomatic infectious period for symptomatic agents), recovered and dead. The infected compartments are further stratified by their presented symptoms, including asymptomatic, presymptomatic, symptomatic mild or severe. All symptomatic agents will also first undergo an infectious presymptomatic period after the exposed latent period. They will either develop mild symptoms who will always recover from the disease or experience severe infection which could either lead to death or recovery. PATAT uses age-structured wild-type SARS-CoV-2 disease severity and mortality probabilities (i.e. $p_{symptomatic}$ = probability of symptomatic disease, p_{severe} = probability of developing severe symptoms, p_{death} = probability of death as a result of COVID-19) as tabulated in Table S1. The probability of developing severe disease is adjusted by a factor of $f_{severe,mt}$ as a result of being infected by the mutant (mt) virus (Table S1). As a simplification, PATAT currently assumes that all agents presenting severe symptoms will be hospitalized and removed from the population.

The total duration of infection since exposure (t) depends on the symptoms presented by the patient and is comprised of different phases (i.e. latent (t_{latent} if individual is symptomatic or $t_{exposed}$ if individual is asymptomatic), asymptomatic ($t_{recovery,asymptomatic}$), presymptomatic ($t_{presymptomatic}$), onset-to-recovery ($t_{onset-to-severe}$, $t_{recovery,mild}$, $t_{recovery,severe}$) and/or death (t_{death})) (Table S1).

Within-host viral dynamics

For each infected agent, PATAT explicitly simulates their viral load trajectory of cycle threshold (Ct) values over the course of their infection using a stochastic model modified from the one previously developed by [2]. A baseline Ct value ($Ct_{baseline}$) of 40 is established upon exposure. The infected agent becomes infectious upon the end of the latent period and their Ct value is assumed to be ≤ 30. A peak Ct value (Ct_{peak}) is then randomly drawn from a truncated normal distribution with the given mean and standard deviation values of the transmitted variant virus (Table S1). The randomly drawn Ct_{peak} must inclusively lie between 1 and 29. Ct_{peak} is assumed to occur upon symptom onset for symptomatic agents and one day after the latent period for asymptomatic individuals. Cessation of viral shedding (i.e. return to $Ct_{baseline}$) occurs upon recovery or death. PATAT assumes that the transition rate towards peak Ct value should not be drastically different to that when returning to baseline upon cessation (i.e. there should be no sharp increase to baseline Ct value after gradual decrease to peak Ct value or vice versa). As such, the time periods of the different phases of infection are randomly drawn from the same quintile of their respective sample distribution. The viral load trajectory is then simulated by

fitting a cubic Hermite spline to the generated exposed (day of exposure, $Ct_{baseline}$), latent (t_{latent}, $Ct_{latent} = 30$), peak (t_{peak}, Ct_{peak}) and cessation values ($t_{onset-to-severe/recovery/death}$, $Ct_{baseline}$). The slope of the fitted curve is assumed to be zero for all of them except during t_{latent} where its slope is assumed to be $\frac{Ct_{peak}-Ct_{baseline}}{t_{peak}-t_{exposed}}$. PATAT then uses the fitted trajectory to linearly interpolate the viral load transmissibility factor ($f_{load,i}$) of an infectious agent i assuming that they are twice as transmissible at peak Ct value (i.e. $f_{load} = 2$) relative to when they first become infectious (i.e. Ct value = 30; $f_{load} = 1$).

Transmissions

When an infectious agent i comes into contact with a susceptible individual j, the propensity of transmission ($p_{transmission,(i,j)}$) is given by following equation from [3]:

$$p_{transmission,(i,j)} = \beta \times \Phi_i \times f_c \times f_{asymp,i} \times f_{load,i} \times f_{immunity,j} \times f_{susceptiblity,j} \times \rho_i \times \rho_j$$

where β is the base transmission probability per contact, Φ_i is the overdispersion factor modelling individual-level variation in secondary transmissions (i.e. superspreading events), f_c is a relative weight adjusting β for the network setting c where the contact has occurred, $f_{asymp,i}$ is the assumed relative transmissibility factor if infector i is asymptomatic, $f_{immunity,j}$ measures the immunity level of susceptible j against the transmitted virus (i.e. $f_{immunity,j} = 1$ if completely naïve; $f_{immunity,j} = 0$ if fully protected), $f_{susceptiblity,j}$ is the age-dependent relative susceptibility of j, ρ_i and ρ_j are the contact rates of infector i and susceptible j respectively.

Φ_i is randomly drawn from a negative binomial distribution with mean of 1.0 and shape parameter of 0.45[4]. As evidence have been mixed as to whether asymptomatic agents are less transmissible, we conservatively assume there is no difference relative to symptomatic patients (i.e. $f_{asymp,i} = 1$). The age-structured relative susceptibility values $f_{susceptiblity,j}$ are derived from odds ratios reported by [5] (Table S1).

β is determined by running initial test simulations with a range of values on a naïve population with no interventions that would satisfy the target basic reproduction number R_0 as computed from the resulting exponential growth rate and distribution of generation intervals [6]. f_c is similarly calibrated during these test runs such that the transmission probabilities in households, workplaces, schools, and all other community contacts are constrained by a relative weighting of 10:2:2:1 [3].

Testing by Ag-RDT

Unlike PCR which is highly sensitive due to prior amplification of viral genetic materials, the sensitivity of Ag-RDT depends on the viral load of the tested patient. While the specificity of Ag-RDT is assumed to be 98.9%, its sensitivity depends on the Ct values of the tested infected agent: Ct > 35 (0%); 35 – 30 (20.9%); 29 – 25 (50.7%); Ct ≤ 24 (95.8%)[7].

Testing by Ag-RDT may either occur via symptomatic testing at healthcare facilities. First, a symptomatic agent may opt to go into self-isolation upon symptom onset prior to being tested, as decided by a Bernoulli trial with probability $p_{self-isolation}$. Regardless if they were self-isolated, after $\tau_{delay,symp-test}$ days from symptom onset, the symptomatic agent may then decide to get tested with a Bernoulli probability of $p_{symp-test}$ that inversely correlates with the distance between the agent's household and the nearest healthcare facility (Table S1). PATAT assumes that agents who have decided against symptomatic testing (i.e. failed Bernoulli trial) or received negative test results will not seek symptomatic testing again.

Isolation and quarantine

We assumed that agents would change their behaviour when (i) they start to present symptoms and go into self-isolation (10% compliance assumed (i.e. $p_{self-isolation} = 10\%$), 71% endpoint adherence[8]); (ii) they test positive and are isolated for 10 days (50% compliance assumed, 86% endpoint adherence[8]); or (iii) they are household members (without symptoms) of positively-tested agents and are required to be in quarantine for 14 days (50% compliance assumed, 28% endpoint adherence[8]). Once an agent goes into isolation/quarantine, we linearly interpolate their probability of adherence to stay in isolation/quarantine over the respective period. Given the lack of infrastructure and resources to set up dedicated isolation/quarantine facilities in many low-middle income countries, we assumed that all isolated and quarantined individuals would do so at home. Although they have no contact with agents outside of their home, we assumed that they would maintain 90% contact rate with household members.

Model Validation

To validate our model, we simulated a 90-day epidemic wave for 1,000,000 individuals using the demography parameters for Zambia (Table S1). We assumed an average of ~40 tests/100,000 people/day were performed during this period. This was the testing rate reported in Zambia between 25 December 2020 and 24 March 2021 when the country was experiencing a second wave of infections as a result of the Beta variant. We assumed the initial effective reproduction rate of ~2.0 and performed 10 independent simulations using PATAT. We then retrieved

confirmed case and death tallies during this period in Lusaka from the Zambia COVID-19 Dashboard (https://www.arcgis.com/apps/dashboards/3b3a01c1d8444932ba075fb44b119b63). We compared our results against data collected in the Lusaka because (1) most of the available COVID-19 incidence data during this period was collected in Lusaka and (2) our model and the demography parameters used best represents urban settings. We multiplied the estimated mean number of reported (i.e. diagnosed) cases and deaths from our simulations by three to proportionally scale our simulation results for three million people, the approximate population size in Lusaka, Zambia. The simulated reported COVID-19 case and death incidence in our model fit well against both actual reported case and death counts respectively (Extended Data Fig. 8).

Background on current guidance

Here, we provide relevant details on the mathematical frameworks underlying three current guidance on minimum sequencing samples required for variant detection mentioned in the main text. Specifically, we highlighted the critical assumptions made and the lack of consideration of spatiotemporal bias resulting from low testing volumes and sampling coverage in each approach.

The World Health Organization (WHO) and European Centre for Disease Prevention and Control (ECDC) computes sequencing sample size using the binomial method.[9,10] **Binomial sampling assumes that specimens to be sampled collected for sequencing are randomly representative of the circulating virus diversity**. As acknowledged by the WHO and ECDC, this is difficult to achieve with low testing rates and spatial non-uniformity in sampling coverage which can introduce spatiotemporal biases in sequencing samples. However, there was no advice in the guidance on how to correct for these biases.

Brito et al. made recommendations on sequencing sample size by computing the probability of detecting at least one variant genome under different sequencing proportions of detected cases based on random subsampling of genomic surveillance data collected in Denmark in 2020-2021[11]. Data from Denmark was used as it was one of the most comprehensive genomic surveillance programs in the world – they were sequencing at >10% of detected cases in most weeks. Brito et al. estimated that sequencing 0.5% of all detected cases would result in sequencing at least one variant genome before the number of variant infections reach 100 cases if turnaround time is kept at 21 days. However, there would only be a 20% probability that this would occur based on their subsampling analyses. More importantly, Denmark was testing at one of the highest rates in the world during this period, performing >2,000 tests/100,000 people/day on average (https://www.finddx.org/covid-19/test-tracker/). **Brito et al., however, did not extend their subsampling analyses on the virus diversity among the detected cases under lower testing rates.** This would have otherwise provided corrections on the suggested sampling proportions under lower testing volumes.

Wohl et al. provided the following derivation to compute the sequencing sample size per unit time (n) for *ongoing* surveillance:[12]

$$n \approx -\frac{\ln\left[1 - \Pr(d \leq t)\right]}{G(t) - G(0)}$$

where $\Pr(d \leq t)$ is the probability of detection on or before time t and $G(t)$ is the cumulative density function that model the growth in *circulating* variant proportion over time. Wohl et al. applied the logistic growth curve function to *circulating* variant proportion growth. As such:

$$G'(t) = \frac{1}{r}\ln\left|a + e^{rt}\right| + C$$

where r is the assumed per unit-time growth rate of the variant and $a = \frac{1}{p_0} - 1$ where p_0 is the initial variant virus proportion.

In this form, Wohl et al. assumes that the *observed* variant proportion in the positive specimens collected perfectly matches the *circulating* variant proportion among the infected population. This is only possible if testing volumes are sufficiently large enough. At the proposed target of 1% *circulating* variant proportion, at least 38,031 tests must be performed in total each day to ensure that the *observed* variant proportion is also at 1% with a margin error of 0.1% at 95% confidence. For ~18 million people in Zambia, this means that the average testing rate be maintained at 212 tests/100,000 people/day. This is ~8 times more than the average LMIC testing rate of 27 tests/100,000 people/day.

To account for likely enriched *observed* variant proportion in the sample pool sent for sequencing, Wohl et al incorporated a correction factor $\frac{C_{Ext}}{C_{Var}}$ to the *circulating* variant proportion such that:

$$G'(t) = \frac{1}{r}\ln\left|\left(\frac{C_{Ext}}{C_{Var}}\right)a + e^{rt}\right| + C$$

$\frac{C_{Ext}}{C_{Var}}$ is the ratio of the coefficient of detection for the extant (C_{Ext}) over that for the variant virus (C_{Var}). C_{Ext} and C_{Var} are essentially the joint conditional probabilities of obtaining a variant and extant virus sequence respectively. For the virus v, the coefficient of detection (C_v) is defined as:

$$C_v = \phi_v \gamma_v [\alpha_v \beta_a + (1 - \alpha_v)\beta_s]$$

where ϕ_v is the sensitivity of the diagnostic test to virus v, γ_v is the probability that the detected infection caused by virus v meets the quality threshold for sequencing (i.e. below the stipulated PCR cycle threshold value), α_v is the probability a person infected with virus v is asymptomatic, β_a and β_s are the probabilities an asymptomatic and symptomatic person infected with SARS-CoV-2 were tested (regardless of which variant they were infected by).

Although β_a and β_s are incorporated in $\frac{C_{Ext}}{C_{Var}}$, the correction factor computes the relative likelihood of detection between the extant and variant virus. In other words, $\frac{C_{Ext}}{C_{Var}}$ **only corrects for biases in the *observed* variant proportion due to relative differences in diagnostic sensitivities, sample qualities and conditional asymptomatic and symptomatic testing probabilities between the two viral variants. It does not factor in distortions in the *observed* variant proportion from other sources of bias, including: (1) stochastic effects arising from low testing volumes and unevenness of daily test stock availability, (2) unconditional probability of asymptomatic testing, and (3) spatial biases when only a subset of samples from sentinel sites are sent for sequencing.**

Emergence of SARS-CoV-2 variants of concern

Here, we briefly recount the emergence of other SARS-CoV-2 variants-of-concern (VOC; i.e. the Alpha, Beta, Gamma and Delta) besides Omicron (i.e. timepoint in collection of the first variant sequence) given the prevailing circumstance, then-level of testing and sequencing performed in the respective countries where they likely first emerged from.

Alpha variant

The Alpha variant was first reported in the UK in early December 2020 after public health agencies investigated the rapid increase in COVID-19 cases in Kent, South East England despite prevailing high levels of non-pharmaceutical interventions[13]. Retrospective analyses found that the first Alpha virus that was later sequenced was collected on 20 September 2020 and phylogenetic analyses estimated the time to most recent common ancestor (TMRCA) of the Alpha lineage to be around the same time[14]. The UK was testing at a mean rate of >300 tests per 100,000 people per day (tests/100k/day) in September 2020[15] and randomly sampling an average of 7.9% cases every week in Kent for sequencing[14]. From on our genomic surveillance simulation results, albeit derived for a Zambian population, we would expect the first Alpha virus variant to be collected for sequencing within one week of its emergence under a random sampling approach at these testing and sequencing rates.

Beta variant

The Beta variant was first reported in South Africa in December 2020 as the country experienced its second wave of SARS-CoV-2 infections. The earliest Beta virus selected for sequencing was collected on 8 October 2020 and the TMRCA was estimated to between July and August 2020[16]. South Africa was still in the midst of the peak of the first wave of infection during the estimated TMRCA period and testing at mean rates of ~60 and ~40 tests/100k/day in July and August 2020 respectively[15]. Only ~0.3% of cases identified in South Africa in July-August 2020 were sequenced and deposited in the GISAID EpiCoV database[17,18]. The estimated ~2-3-month delay in sampling the first Beta variant sequence is therefore likely based on our simulations due to a combination of relatively low levels of testing and sequencing that was further exacerbated by the variant virus emerging during the peak circulation of the extant SARS-CoV-2 wild-type virus.

Gamma variant

The Gamma variant was first reported in January 2021 in Brazil as a result of investigating the rapid rise in hospitalizations in Manaus in December 2020[19] as well as in Japan from infected travelers who recently returned from the Amazonas[20]. The first Gamma variant virus selected for sequencing was collected on 6 December 2020 and phylogenetic analyses estimated that the VOC lineage likely emerged in Manaus between October and November 2020 [19]. During this period, Brazil was testing at 10-30 tests/100k/day on average[15] and sequenced ~0.1% of all confirmed cases[17,18]. By early January 2021 when the sequencing results were obtained and shared, the circulating proportion of the Gamma variant in Manaus was estimated to be ~75%[19]. The 1-2 month gap between emergence and sampling of a VOC sequence is likely due to low testing and sequencing rates, consistent with the results of our simulations. Moreover, the turnaround time between sample collection and sequencing data acquisition added an additional month in delay before the Gamma variant was first reported in Brazil.

Delta variant

The earliest Delta (i.e. PANGO lineage B.1.617.2) sequence (Accession: EPI_ISL_9232357) collected in India that is deposited in the GISAID EpiCoV database was collected on 3 September 2020. This sample however was only sequenced retrospectively as (i) it was submitted to the database on 28 January 2022 and (ii) published works by the Indian SARS-CoV-2 Genomics Consortium (INSACOG), the national sentinel sequencing network, referred to earliest identification of Delta in state of Maharashtra in December 2020[21,22]. The identification of Delta in Maharashtra was only done so retrospectively to investigate the surge in cases in the state in January 2021. Using Delta sequences collected globally, the likely TMRCA period was estimated to be around September 2020 as well[23]. During this time, India was still experiencing

the peak of its first wave of SARS-CoV-2 infections[18]. The second wave of SARS-CoV-2 infections across the country caused by the Delta variant only took off in March 2021, six months after the estimated TMRCA[24]. Between December 2020 when the first wave of infections subsided and the beginning of the second wave in March 2021, there were several competing lineages circulating in India, including the Alpha VOC as well as the Kappa variant of interest (i.e. B.1.617.1), a sub-lineage descending from the same parental lineage of Delta (i.e. B.1.617)[24]. There were no coordinated efforts to perform active genomic surveillance across India in 2020; Sequencing analyses then were largely performed retrospectively in response to surge in cases[22,25]. The INSACOG was only established by the government on 30 December 2020 in response to monitor genetic variations in light of the introduction of the Alpha variant into the country[26,27]. Owing to complexities attributed to multiple co-circulating and competing variant lineages, nonuniformity in sampling, in part due to a lack of ongoing active coordinated nation-wide genomic surveillance efforts then, and that the "earliest" Indian Delta sequences were all identified from retrospective analyses, there are uncertainties around both the emergence and early spread of the Delta variant within India.

Supplementary Tables

Supplementary Table 1. PATAT simulation parameters.

Parameter	Values/Distribution	Source
Population demography		
Total population size	1,000,000	
Mean household size	5.0	[28]
Age structure (in bins of 5 years)	[0.161, 0.165, 0.157, 0.101, 0.083, 0.068, 0.057, 0.051, 0.042, 0.030, 0.024, 0.015, 0.016, 0.009, 0.008, 0.005, 0.006, 0.002, 0.000, 0.000]	[28]
Minimum prime adult age	20 years	Assumed
Proportion of women	51%	[29]
Minimum working age	15 years	[29]
Employment rate	39% (male), 23% (female)	[29]
Formal employment rate	36% (employed male), 24% (employed female)	[29]
Schooling rate	79% (male), 40% (female)	[28]
School gender parity	1.0 (Primary), 0.9 (Secondary)	[28]
Church participation rate	70% of all households	Assumed
Mean employment contacts (formal)	20	Assumed
Mean employment contacts (informal)	5	Assumed
Mean class size	37 (Primary and secondary)	[28]
Mean school size	700 (Primary and secondary)	Assumed
Student-teacher ratio	42 (Primary and secondary)	[28]
Mean church size (s.d.)	500 (100)	Assumed
Mean random contacts in church per person	10	Assumed

Mean random community contacts per day	10	Assumed
SARS-CoV-2 transmissions related parameters		
Age-structured relative susceptibility (in bins of 5 years, $f_{susceptiblity}$)	[0.34, 0.34, 0.67, 0.67, 1.00, 1.00, 1.00, 1.00, 1.00, 1.00, 1.00, 1.00, 1.00, 1.00, 1.24, 1.24, 1.47, 1.47, 1.47, 1.47]	[3,5]
Age-structured probability of becoming symptomatic (in bins of 5 years, $p_{symptomatic}$)	[0.50, 0.50, 0.55, 0.55, 0.60, 0.60, 0.65, 0.65, 0.70, 0.70, 0.75, 0.75, 0.80, 0.80, 0.85, 0.85, 0.90, 0.90, 0.90, 0.90]	[30,31]
Age-structured probability of developing severe disease (in bins of 5 years, p_{severe})	[0.00050, 0.00050, 0.00165, 0.00165, 0.00720, 0.00720, 0.02080, 0.02080, 0.03430, 0.03430, 0.07650, 0.07650, 0.13280, 0.13280, 0.20655, 0.20655, 0.24570, 0.24570, 0.24570, 0.24570]	[30,31]
Age-structured probability of death (in bins of 5 years, p_{death})	[0.00002, 0.00002, 0.00002, 0.00002, 0.00010, 0.00010, 0.00032, 0.00032, 0.00098, 0.00098, 0.00265, 0.00265, 0.00766, 0.00766, 0.02439, 0.02439, 0.08292, 0.08292, 0.16190, 0.16190]	[8,32]
Latent period (days, t_{latent})	Wild-type SARS-CoV-2/Alpha: Lognormal (4.5, 1.5)<br>Delta/Omicron: Lognormal (4.0, 1.3)	[3,33–35]
Pre-symptomatic period (days, $t_{presymptomatic}$)	Wild-type SARS-CoV-2/Alpha: Lognormal (1.1, 0.9)<br>Delta/Omicron: Lognormal (1.8, 1.7)	[3,33,35]
Period between symptom onset and severe disease (days, $t_{onset-to-severe}$)	Lognormal (6.6, 4.9)	[33]
Period between severe disease and death (days, t_{death})	Lognormal (8.6, 6.7)	[33]
Recovery period for symptomatic agents with mild disease (days, $t_{recovery,mild}$)	Wild-type SARS-CoV-2/Alpha: Lognormal (8.0, 2.0)<br>Delta: Lognormal (6.23, 0.53*)<br>Omicron: Lognormal (5.35, 0.37*)	[35,36]
Recovery period for asymptomatic agent (days, $t_{recovery,asymptomatic}$)	Wild-type SARS-CoV-2/Alpha: Lognormal (8.0, 2.0)<br>Delta: Lognormal (6.23, 0.53*)<br>Omicron: Lognormal (5.35, 0.37*)	[35,36]
Recovery period of agents with severe disease (days, $t_{recovery,severe}$)	Lognormal (18.1, 6.3)	[30]
Peak Ct values (Ct_{peak})	Wild-type SARS-CoV-2/Alpha/Delta: Normal (20.5, 0.79*)	[35]

	Omicron: Normal (23.3, 0.58*)	
Cross-immunity to variant virus after infection by extant virus (i.e. $1 - f_{immunity}$)	Wild-type SARS-CoV-2/Alpha: 0.87<br>Delta/Omicron: 0.20	[37,38]
Severity (chance of hospitalization) of variant relative to extant virus ($f_{severe,mt}$)	Wild-type SARS-CoV-2/Alpha: 100%<br>Delta/Omicron: 40%	[39]
Testing parameters		
Delay in visiting healthcare facility for symptomatic testing (days, $\tau_{delay,symp-test}$)	Lognormal (1.0, 0.5)	Assumed
Agents to healthcare facilities ratio	7,000:1	[40,41]
Distance-structured distribution of households to nearest healthcare facility (in bins of 1km)	[0.048, 0.193, 0.119, 0.08, 0.074, 0.098, 0.068, 0.072, 0.056, 0.191]	[42]
Distance-structured probabilities of agent visiting nearest healthcare facility for testing services (in bins of 1km, $p_{symp-test}$)	[0.853, 0.808, 0.762, 0.717, 0.672, 0.626, 0.581, 0.536, 0.49, 0.445]	[42]
Isolation/quarantine parameters		
Isolation period	10 days	
Quarantine period	14 days	
Self-isolation period	10 days	
Reduction in contact rates under isolation/quarantine (in order of households, schools, workplaces, church and random community)	[10%, 100%, 100%, 100%, 100%]	

*Standard deviation values inferred from 95% confidence interval computed in reference.

References

1. Pew Research Center. The Age Gap in Religion Around the World. https://www.pewresearch.org/religion/2018/06/13/the-age-gap-in-religion-around-the-world/ (2018).

2. Quilty, B. J. *et al.* Quarantine and testing strategies in contact tracing for SARS-CoV-2: a modelling study. *Lancet Public Health* **6**, e175–e183 (2021).

3. Kerr, C. C. *et al.* Covasim: An agent-based model of COVID-19 dynamics and interventions. *PLoS Comput Biol* **17**, e1009149- (2021).

4. Endo, A., Abbott, S., Kucharski, A. J. & Funk, S. Estimating the overdispersion in COVID-19 transmission using outbreak sizes outside China. *Wellcome Open Research* **5**, 67 (2020).

5. Zhang, J. *et al.* Changes in contact patterns shape the dynamics of the COVID-19 outbreak in China. *Science* **368**, 1481–1486 (2020).

6. Wallinga, J. & Lipsitch, M. How generation intervals shape the relationship between growth rates and reproductive numbers. *Proceedings of the Royal Society B: Biological Sciences* **274**, 599–604 (2006).

7. Brümmer, L. E. *et al.* Accuracy of novel antigen rapid diagnostics for SARS-CoV-2: A living systematic review and meta-analysis. *PLoS Med* **18**, e1003735 (2021).

8. Brazeau, N. F. *et al.* Report 34 - COVID-19 Infection Fatality Ratio Estimates from Seroprevalence. (2020) doi:10.25561/83545.

9. World Health Organization. Guidance for surveillance of SARS-CoV-2 variants: interim guidance, 9 August 2021. https://apps.who.int/iris/handle/10665/343775 (2021).

10. European Centre for Disease Prevention and Control. Sequencing of SARS-CoV-2 - first update. https://www.ecdc.europa.eu/en/publications-data/sequencing-sars-cov-2 (2022).

11. Brito, A. F. *et al.* Global disparities in SARS-CoV-2 genomic surveillance. Preprint at *medRxiv* https://doi.org/10.1101/2021.08.21.21262393 (2021).

12. Wohl, S., Lee, E. C., DiPrete, B. L. & Lessler, J. Sample Size Calculations for Variant Surveillance in the Presence of Biological and Systematic Biases. Preprint at *medRxiv* https://doi.org/10.1101/2021.12.30.21268453 (2022).

13. UK Health Security Agency. Investigation of SARS-CoV-2 variants of concern: technical briefings. https://www.gov.uk/government/publications/investigation-of-novel-sars-cov-2-variant-variant-of-concern-20201201 (2022).

14. Hill, V. *et al.* The Origins and Molecular Evolution of SARS-CoV-2 Lineage B.1.1.7 in the UK. *Virus Evol* **8**, veac080 (2022).

15. FIND. Test tracker - FIND. https://www.finddx.org/covid-19/test-tracker/ (2022).

16. Tegally, H. *et al.* Detection of a SARS-CoV-2 variant of concern in South Africa. *Nature* **592**, 438–443 (2021).

17. GISAID. GISAID - Initiative. https://www.gisaid.org/.

18. Johns Hopkins University Center for Systems Science and Engineering. CSSEGISandData/COVID-19: Novel Coronavirus (COVID-19) Cases. https://github.com/CSSEGISandData/COVID-19.

19. Faria, N. R. *et al.* Genomics and epidemiology of the P.1 SARS-CoV-2 lineage in Manaus, Brazil. *Science* **372**, 815-821 (2021).

20. Fujino, T. *et al.* Novel SARS-CoV-2 Variant in Travelers from Brazil to Japan. *Emerg Infect Dis* **27**, 1243–1245 (2021).

21. Mlcochova, P. *et al.* SARS-CoV-2 B.1.617.2 Delta variant replication and immune evasion. *Nature* **599**, 114–119 (2021).

22. Cherian, S. *et al.* SARS-CoV-2 Spike Mutations, L452R, T478K, E484Q and P681R, in the Second Wave of COVID-19 in Maharashtra, India. *Microorganisms* **9**, 1542 (2021).

23. McCrone, J. T. *et al.* Context-specific emergence and growth of the SARS-CoV-2 Delta variant. *Nature* **610**, 154-160 (2022).

24. Dhar, M. S. *et al.* Genomic characterization and epidemiology of an emerging SARS-CoV-2 variant in Delhi, India. *Science* **374**, 995–999 (2021).

25. Srivastava, S., Banu, S., Singh, P., Sowpati, D. T. & Mishra, R. K. SARS-CoV-2 genomics: An Indian perspective on sequencing viral variants. *J Biosci* **46**, 22 (2021).

26. Thiagarajan, K. Why is India having a covid-19 surge? *BMJ* **373**, n1124 (2021).

27. Ministry of Health and Family Welfare. Q&A on Indian SARS-CoV-2 Genomics Consortium (INSACOG). https://dbtindia.gov.in/pressrelease/qa-indian-sars-cov-2-genomics-consortium-insacog.

28. Zambia Statistics Agency. Zambia Demographic and Health Survey 2018. https://www.zamstats.gov.zm/index.php/publications/category/8-demorgraphy?download=364:zambia-demographic-and-health-survey-2018 (2018).

29. Zambia Statistics Agency. 2019 Labour Force Survey Report. https://www.zamstats.gov.zm/index.php/publications/category/7-labour (2019).

30. Verity, R. *et al.* Estimates of the severity of coronavirus disease 2019: a model-based analysis. *Lancet Infect Dis* **20**, 669–677 (2020).

31. Ferguson, N. M. *et al.* Report 9: Impact of non-pharmaceutical interventions (NPIs) to reduce COVID-19 mortality and healthcare demand. (2020) doi:10.25561/77482.

32. O'Driscoll, M. *et al.* Age-specific mortality and immunity patterns of SARS-CoV-2. *Nature* **590**, 140–145 (2020).

33. Linton, N. M. *et al.* Incubation Period and Other Epidemiological Characteristics of 2019 Novel Coronavirus Infections with Right Truncation: A Statistical Analysis of Publicly Available Case Data. *Journal of Clinical Medicine* **9**, 538 (2020).

34. Kang, M. *et al.* Transmission dynamics and epidemiological characteristics of SARS-CoV-2 Delta variant infections in Guangdong, China, May to June 2021. *Euro Surveill* **27**, 2100815 (2022).

35. Hay, J. A. *et al.* Viral dynamics and duration of PCR positivity of the SARS-CoV-2 Omicron variant. Preprint at *medRxiv* https://doi.org/10.1101/2022.01.13.22269257 (2022).

36. Wölfel, R. *et al.* Virological assessment of hospitalized patients with COVID-2019. *Nature* **581**, 465–469 (2020).

37. Pouwels, K. B. *et al.* Effect of Delta on viral burden and vaccine effectiveness against new SARS-CoV-2 infections in the UK. *Nat Med* **27**, 2127-2135 (2021).

38. Imperial College London. Report 49 - Growth, population distribution and immune escape of Omicron in England. https://www.imperial.ac.uk/mrc-global-infectious-disease-analysis/covid-19/report-49-Omicron/ (2021).

39. Imperial College London. Report 50 - Hospitalisation risk for Omicron cases in England. https://www.imperial.ac.uk/mrc-global-infectious-disease-analysis/covid-19/report-50-severity-omicron/ (2022).

40. Nichols, B. E. *et al.* Monitoring viral load for the last mile: what will it cost? *J Int AIDS Soc* **22**, e25337 (2019).

41. Girdwood, S. J. *et al.* Optimizing viral load testing access for the last mile: Geospatial cost model for point of care instrument placement. *PLoS One* **14**, e0221586 (2019).

42. Dovel, K. *et al.* Frequency of visits to health facilities and HIV services offered to men, Malawi. *Bull World Health Organ* **99**, 618–626 (2021).
